# Uplift resistance capacity of anchor piles used in marine aquaculture

**DOI:** 10.1038/s41598-021-99817-5

**Published:** 2021-10-13

**Authors:** Fukun Gui, Jianqiao Kong, Dejun Feng, Xiaoyu Qu, Fang Zhu, Yang You

**Affiliations:** 1 National Engineering Research Center for Marine Aquaculture, Zhejiang Ocean University, Zhoushan, 316022 China; 2 School of Fisheries, Zhejiang Ocean University, Zhoushan, 316022 China

**Keywords:** Physical oceanography, Civil engineering

## Abstract

Anchor piles are widely used in marine aquaculture, and the safety is largely determined by the uplift resistance capacity,especially in harsh ocean environments. However, there are few practical guides to the design and installation of the anchor piles for mooring the body of marine aquaculture equipment. Laboratory experiments were conducted to investigate the effect of the initial tension angle, pile diameter, embedded depth, and pile configuration on the uplift resistance capacity of anchor piles under oblique loads. CCD camera and load cell were utilized to measure the corresponding displacement and load, respectively. The results show that increasing the initial tension angle of circular and square single piles can significantly improve the uplift resistance capacity. The failure load of the square single pile was slightly higher than that of the circular single pile. Increasing the pile diameter can effectively improve the failure load and delay the development speed of the pile top displacement. Increasing the embedded depth can effectively improve the failure load and increase the lateral displacement of the pile top. The uplift resistance capacity of the dual anchor piles was better than that of the single anchor piles. The layout configuration has little effect on the failure load, but has a large effect on the displacement development.

## Introduction

**Figure 1:**
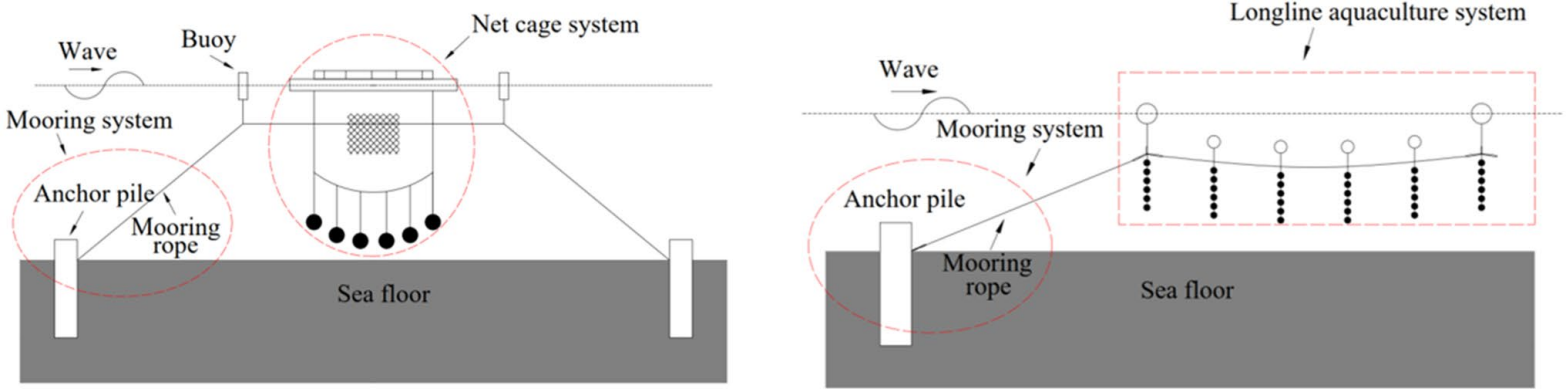
Marine aquaculture system: (**a**) net cage aquaculture; (**b**) longline aquaculture.

Net cage and longline aquaculture are two main marine aquaculture methods and the hydrodynamic characteristics of the floating parts have been studied comprehensively^1–4^. There are, however, limited studies that focused on the anchor piles, which are driven by oblique tension load and widely used in the mooring system of marine aquaculture equipment (Fig. 1). Trujillo et al.^5^ use scale models investigating the behavior of five anchoring and use one “rezon” type deadweight designs, determining their uplift performance in the sand. Cortes-Garcia et al.^6^ 3D finite element analyses to illustrating the capacity of helical anchors in an estuarine environment for aquaculture. Hou et al.^7^ has been utilized the Lumped-mass method to investigate the dynamic response of mooring line considering the influence of embedded chains in clay for a net cage system. The study of the uplift resistance of anchors is well established in another field of engineering^8–11^. However, the most of the anchors mentioned are used in offshore structures such as offshore drilling platforms, and the designed water depth is not less than 100 m. The mainly designed water depth of marine aquaculture engineering equipment is generally within 50 m^12^, and the above research results cannot be directly applied to marine aquaculture engineering equipment.

Previous studies on pile foundations that provided support for cross-sea bridges, ocean platform and other ocean structures can shed light on the behavior of anchor piles have more studies under oblique loads. Ayothiraman et al.^13^ and Reddy et al.^14^ conducted uplift laboratory model experiments with and without a lateral load found that the failure load increased in the case of lateral load, and the displacement and deflection of the pile top also increased significantly. This simplified analysis method does not fully consider the coupling effect of combined loads. Lu et al.^15^ applied the oblique load directly in the model pile and the results showed that “soil densification effect” and “P-$\Delta$ effect” emerged from the tests. Shin et al.^16^ obtained an empirical formula for the relationship between ultimate bearing capacity and load inclination by laboratory model tests on uplifted piles subjected to inclined loads in saturated cohesive clay. Yang et al.^17^ found that after the pile is failed by the oblique load, the clay within a certain depth of its pulled side will be destroyed and the damage area is fan-shaped. Ramadan et al.^18,19^ used centrifuge tests and numerical simulation software to study the uplift resistance of anchor piles in sand. The changes of bending moment and earth pressure during the uplift process of anchor piles under variables tension angle have been analyzed. Although the behaviours of pile foundation and anchor pile are not exactly the same, the related studies on pile foundation can provide useful information on the experimental methodology.

This study investigates the uplift resistance capacity of the marine aquaculture anchor pile with different cases by means of a physical model experiment. A series of model tests were conducted with the main objective of investigating the effect of the initial tension angle, pile diameter, embedded depth, and pile configuration on the uplift resistance to provide a practical guide for the application of anchor piles in marine aquaculture.

## Materials and methods

### Test system

**Figure 2:**
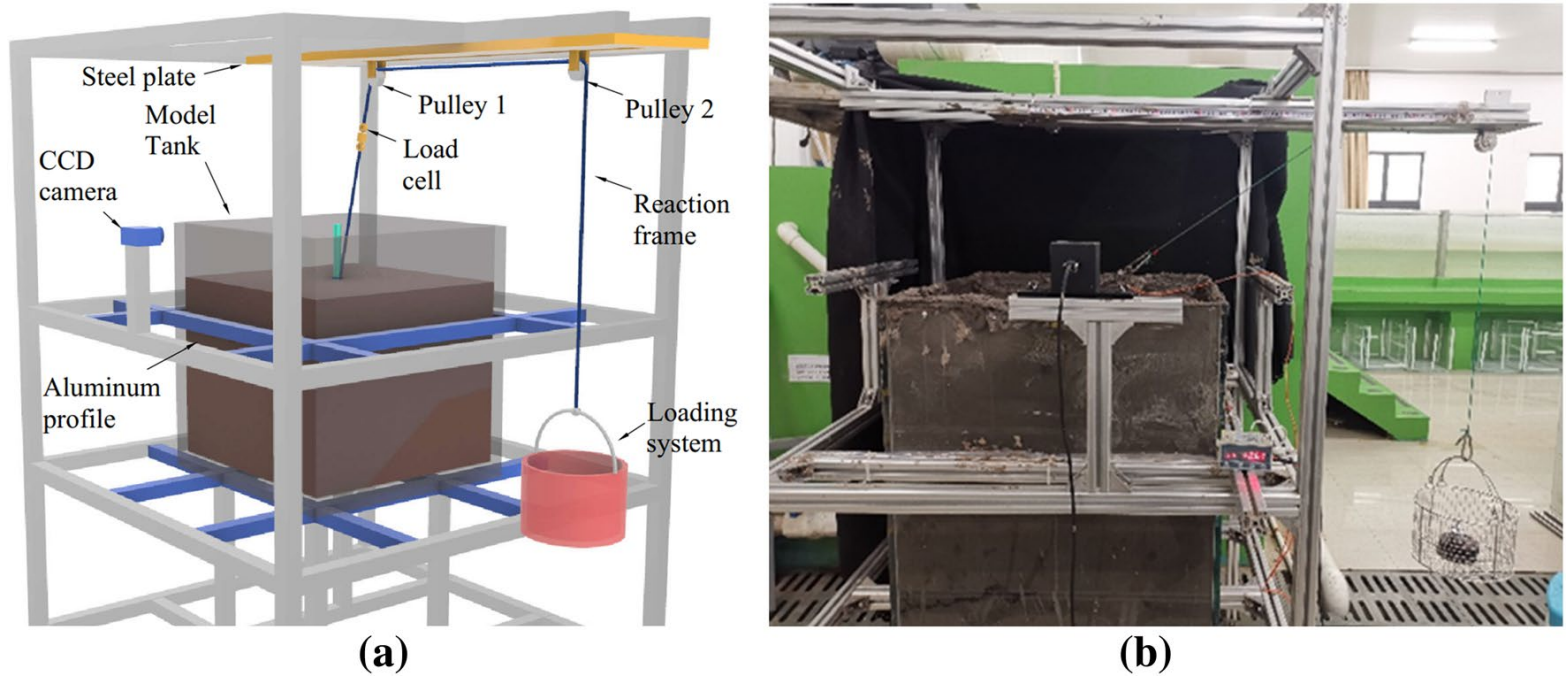
Schematic diagram (**a**) and photo (**b**) of the experimental setup.

The experiment was conducted at the Marine Engineering Hydrodynamics Laboratory of the National Engineering Research Center for Marine Aquaculture, China. The test system consisted of four parts as shown in Fig. 2a: a reaction frame, model tank, uplift system, and loading system. The reaction frame was constructed of aluminum profiles (40 mm × 40 mm cross-sectional size), on which all the experimental devices used were installed. The model tank (700 mm × 700 mm × 700 mm, length width height) made of glass (15 mm, thickness), was filled with clay, in which an anchor pile model was installed in the center. The distance from the center of the model tank to the inner wall of the tank was 335 mm, which was greater than 10 times the maximum diameter of the anchor pile model of 30 mm, satisfying the boundary effect^20,21^. The uplift system consisted of a double-stranded twine rope with a diameter of 2 mm and two pulleys that were fixed on a steel plate. The steel plate was positioned 0.5 m above the tank. The loading system consisted of a loading box and weights. The rope was connected to the anchor pile model and the loading system after passing through the pulleys. As shown in Fig. 2a, a load cell (Bengbu Dayang Sensor Co., Ltd., range: 0-100 N, accuracy: 0.3$\%$) was installed between pulley 1 and the anchor pile model is used to monitor the load in the rope. A charge-coupled device (CCD) camera (Qingdao Optical Flow Software Technology Co., Ltd., size: 2560 × 2048 pixels) was positioned outside of the model tank to capture the position information of the pile top of the anchor pile model under variables tension load, as shown in Fig. 2b.

### Anchor pile model

**Figure 3:**
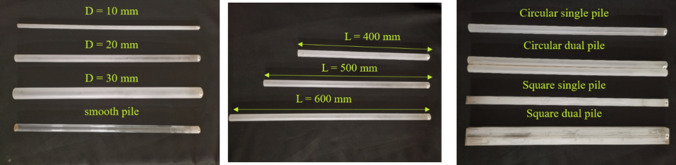
Anchor pile models with variables in diameter (**a**), length (**b**) and configuration (**c**).

**Figure 4:**
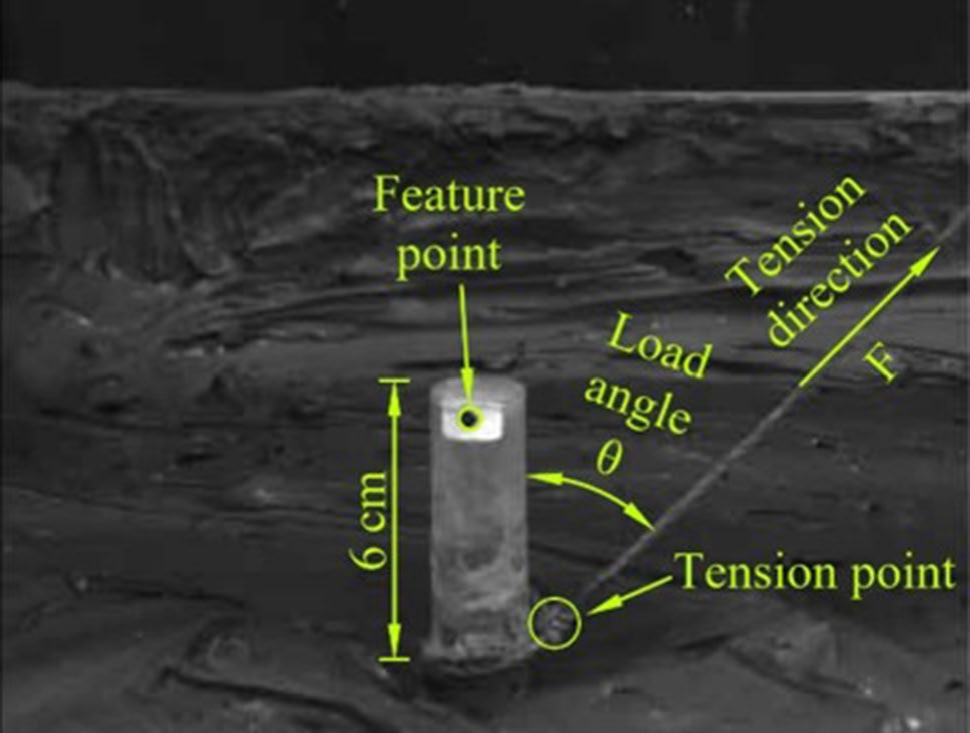
Installation of the model anchor pile.

The anchor pile model was mainly driven by oblique uplift force and the geometric and dynamic similarities should be considered seriously when designing the models^22^. The primary parameters of the prototype and model must satisfy the following relationships:

1 $$\begin{aligned} C_L&=\frac{L_P}{L_M} \end{aligned}$$

2 $$\begin{aligned} E_M I_M&= \frac{1}{C_L^{5} } E_{P} I_{P} \end{aligned}$$

where $L_P$, $E_P$ , and $I_P$ are the characteristic length, elastic modulus, and moment of inertia of the prototype, respectively, and $L_M$,$E_M$, and $I_M$ are the characteristic length, elastic modulus, and moment of inertia of the model, respectively. Thus, $C_L$ is a scale factor. The material of the prototype anchor pile is assumed to be steel and has following, parameters, pile diameter D = 140 mm, wall thickness t = 5 mm, and bending stiffness *EI* = 205 GPa. Based on the similarity law, the scale factor is set to be $C_L$ is 1:7, the plexiglass with a thickness of 2 mm was used to make the pile models. The flexural rigidity of the anchor pile model with a diameter of 20 mm measured by a simple cantilever beam test was 136.2 Pa. Hereinafter, anchor piles constructed of two pipes are referred as the dual pile. The model used in the test is shown in Fig. 3a–c. To model the friction between the anchor pile and the clay, the surface of the anchor pile model was sandblasted with quartz sand (180 mesh) and the anchor pile model without sandblasting also was shown in Fig. 3a for comparison. In this study the tension point of the anchor pile model is uniformly set on the surface of the clay, 6 cm from the top of the model, as shown in Fig. 4.

### Clay

The marine clay used in the study was collected from Changzhi Island, Zhejiang Province, China. Before starting the experiment, the clay was sieved to remove internal impurities. And then water was added and stirred to make it fully saturated. The specific parameters of the remoulded clay are presented in Table 1 and the clay was considered as silty clay.

**Table 1:** Parameters of the clay.

Density $\rho$ (g $\cdot$ cm$^{-3}$)	Proportion $G_s$	Void ratio $e_0$	Moisture content $\omega$ ($\%$)	Liquid limit $\omega _L$ ($\%$)	Plastic limit $\omega _P$ ($\%$)
1.67	2.73	1.555	56.3	40.2	22.5

### Test procedure

To systematically study the uplift resistance capacity of anchor piles used in marine aquaculture, six test cases were designed and listed in Table 2. Here, D is the pile diameter, L is the embedded depth, and $\theta$ is the initial tension angle. The main experimental steps were as follows: (1) Move the remoulded clay into the tank and stir continuously to keep it well-distributed. Then keep the clay still for one week to make it compact. (2) Install the anchor pile model at the designed depth of the clay with care to avoid disturbing the clay around the model. (3) Increase the load by adding calibration weights with an increase about 1/10 of the estimated failure load gradually and record load and the position information of the top of the anchor pile. (4) When the anchor piles reach the failure criteria, take out the model and recover the clay and then start the next set of the experiments.

**Table 2:** Experimental conditions.

Case	Pile type	Diameter D (mm)	Embedded depth L (mm)	Initial tension angle $\theta$ (°)	Layout angle (°)
1	Circular single pile	20	440	0, 15, 30, 45, 60	–
2	Square single pile	20 (side length)	440	0, 15, 30, 45, 60	–
3	Circular single pile	10, 20, 30	440	30, 45, 60	–
4	Circular single pile	20	340, 440, 540	30, 45, 60	–
5	Circular dual pile	–	440	30, 45, 60	0,90
6	Square dual pile	–	440	30, 45, 60	0,90

### Anchor pile failure criteria

Under the oblique load condition, the displacement–load curve usually has not exhibited a distinct plunge point^23^. Therefore, it is of vital importance to adopt the failure criteria to evaluate the uplift resistance of the anchor piles. However, there are no widely-acknowledged failure load criteria for anchor piles in marine aquaculture^17,23–25^. In addition, Cortes-Garcia et al.^6^ showed that the failed criteria for onshore structures is too strict for offshore structures. Therefore, relatively moderate failure criteria should be formulated for the anchor piles used in marine aquaculture. Considering the above theory and the experimental conditions, the following anchor pile failure criteria were defined. 1.Under the action of one stage load, the vertical or lateral displacement of the pile top increased suddenly. 2.The cumulative vertical displacement of the pile top is greater than 0.5 D. 3.The cumulative lateral displacement of the pile top was greater than 1 D.

### Data processing

In this experiment, the displacement of the top of the anchor pile was used to evaluate the corresponding uplift resistance capacity. A black circular paper was attached to the top of the anchor pile model as a feature texture to ensure that the CCD camera can accurately capture the position information of the anchor pile model. The recorded images were processed in a way of cross correlation algorithm as that in PIV (Particle Image Velocimetry). Length calibration should be done firstly to figure out the relationship between image pixel and physical length. Then, two adjacent images were processed by cross correlation algorithm to find the peak correlation coefficient of the feature point in the second image to locate its position. Therefore, the displacement in the two images were successfully extracted and the time history of the displacement of the pile top can be obtained and used for further analysis.

## Results and discussion

### Effect of initial tension angle on the uplift resistance capacity

The initial tension angle is a key factor that must be considered during the installation of the anchor pile. This study (Cases 1 and 2)investigated the effect of the initial tension angle on the uplift resistance capacity.

**Figure 5:**
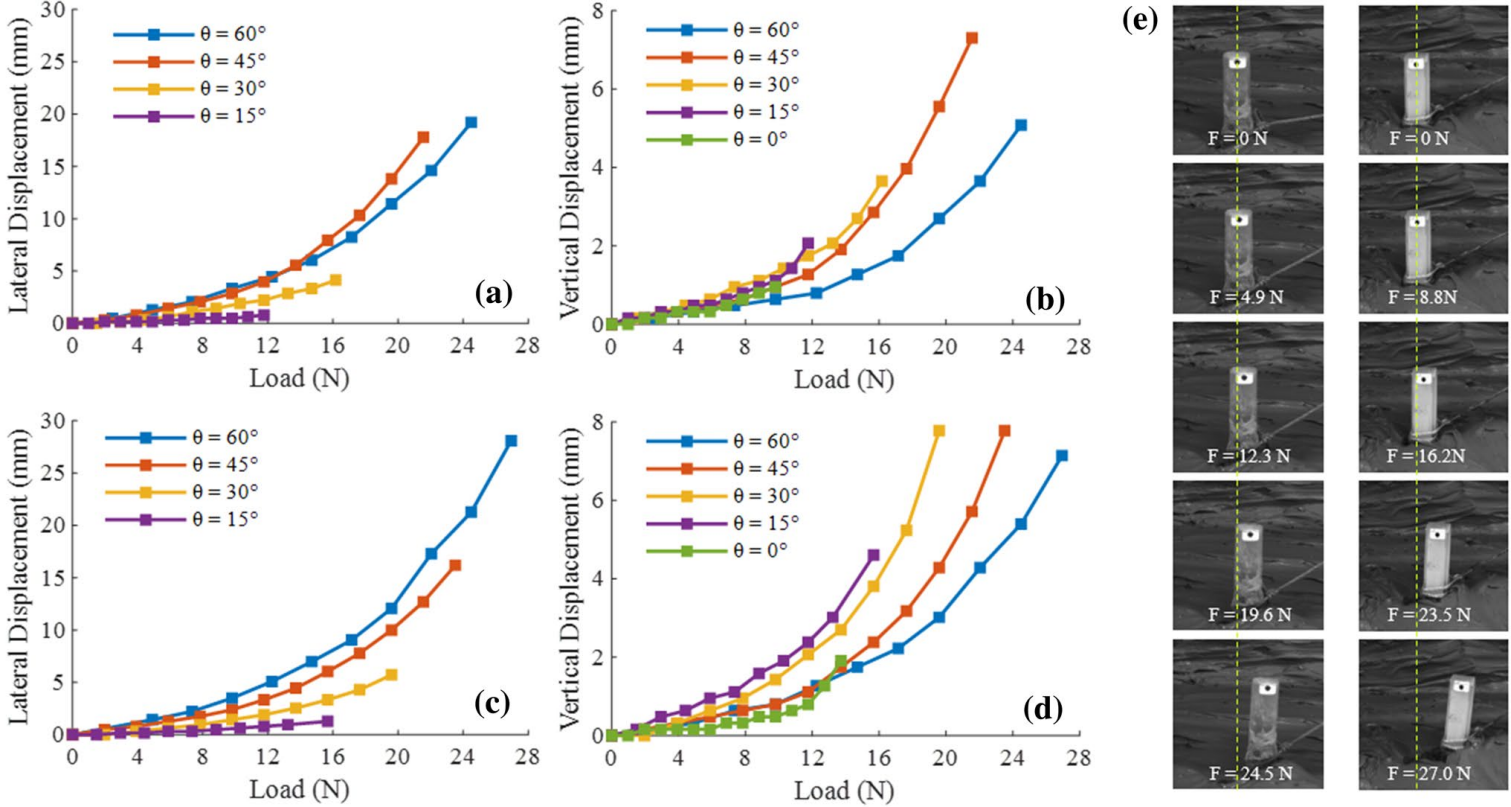
Lateral and vertical displacement–load curves with variables angle of circular pile (**a**,**b**) and square pile (**c**,**d**), and the failure process of circular pile (left column) and square pile (right column) with $\theta$ = 60° (**e**).

Figure 5a–d shows the pile top displacement–load curves of anchor piles under variables initial tension angle. This study focuses on analyzing the data from the beginning of loading to the failure of the anchor pile. As shown in Fig. 5a–d, except for $\theta$ = 0° ($\theta$ is the initial angle of tension), other initial tension angle cases have lateral displacements. The initial tension angle has a significant effect on the top displacement of the circular and square single piles. For circular single piles, when the initial tension angle is small ($\theta$ = 0°, 15°, 30°), as the load increases, the lateral and vertical displacements of the pile top slowly increase. The failure load was small, and both the lateral and vertical displacements were small. When the initial tension angle increased ($\theta$ = 45°, 60°), as the load increased, the failure load and the maximum lateral and vertical displacements were all relatively large. For square single piles, under the same initial tension angle cases, the square and circular single pile tops have similar trends in the change of displacement.

**Figure 6:**
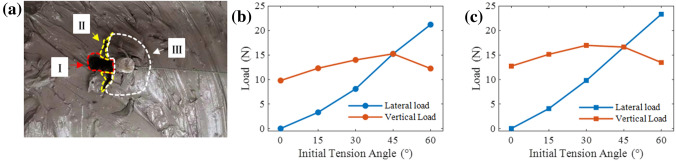
Photo of clay deformation (circular single pile, $\theta$ = 60°) before failure (**a**). Relationship between the lateral and vertical components of anchor pile failure load with the initial tension angle circular single pile (**b**) and square single pile (**c**).

Figure 5e is a photograph of the failure process of the anchor pile model under $\theta$ = 60°, where the left and right panels are for a circular and square pile, respectively. The yellow dotted line indicates the initial position of the marked feature point on the top of the anchor pile model, and F is the load on the anchor pile. Table 3 summarizes the maximum displacements of the circular and square single piles under variables initial tension angle. The maximum total displacement of $\theta$ = 45° and $\theta$ = 60° was much larger than that of the other cases. In Fig. 6a, the red area *I* is the clay deformation caused by the lateral displacement of the anchor pile. The yellow area *II* is due to insufficient cohesion between the clay around the pile and the failure cracks caused by the deformation of the clay. The white area *III* is due to the accumulation of clay after deformation.

**Table 3:** Maximum displacement of pile top under variables initial tension angle.

Pile type	Maximum displacement (unit: mm)	0°	15°	30°	45°	60°
Circular single pile	Lateral	–	1.11	4.13	17.78	19.21
	Vertical	0.95	2.06	3.65	7.30	5.08
	Total	0.95	2.34	5.51	19.22	19.87
Square single pile	Lateral	–	1.27	5.71	16.19	28.10
	Vertical	1.90	4.60	7.78	7.78	7.14
	Total	1.90	4.77	9.65	17.96	28.99

In addition, the failure load of the square single pile under the same initial tension angle was larger than that of the circular single pile. Because the cross-sectional area of a square with a side length of 20 mm is larger than that of a circular cross-sectional area of 20 mm. The contact area between the square single pile and the clay was larger, the clay provided more reaction force, thereby increasing the failure load.

The literature^9,25–28^ showed that when $\theta>$ 0°, the failure load and initial tension angle satisfy the following elliptic equation:

3 $$\begin{aligned} \left( \frac{F\sin \theta }{F_h}\right) ^{2} + \left( \frac{F\cos \theta }{F_v}\right) ^{2} = 1 \end{aligned}$$

where *F* is the resultant force acting on the structure; $F_h$ and $F_v$ are the lateral and vertical failure loads, respectively; and $\theta$ is the initial tension angle. It can be seen from equation 3 that the existence of a lateral load reduces the vertical uplift resistance. Figure 6b,c shows the relationship between the lateral and vertical components of the anchor pile failure load and the initial tension angle. The decomposition of the failure load is based on the initial tension angle. It should be noted that it would introduce error due to the time-varying tension angle. However, it was found that the difference between the time-varying tension angle and the initial tension angel was less than 1 even at the timing of the maximum displacement of the monopile failure occurred (square anchor pile, $\theta$=60°). In addition, it was not easy to accurately measure the time-varying tension angle. Therefore, the initial tension angle was used to decompose the failure load. The figure shows that with the increase of the initial tension angle, the lateral component of the anchor pile failure load gradually increases, and the vertical component initially increases and then decreases. This shows that the existence of the lateral force component in this experiment improves the vertical uplift resistance capacity of the anchor pile, thereby increasing the failure load of the anchor pile, which conforms to the theory proposed research results^29,30^.

### Effect of pile diameter on the uplift resistance capacity

**Figure 7:**
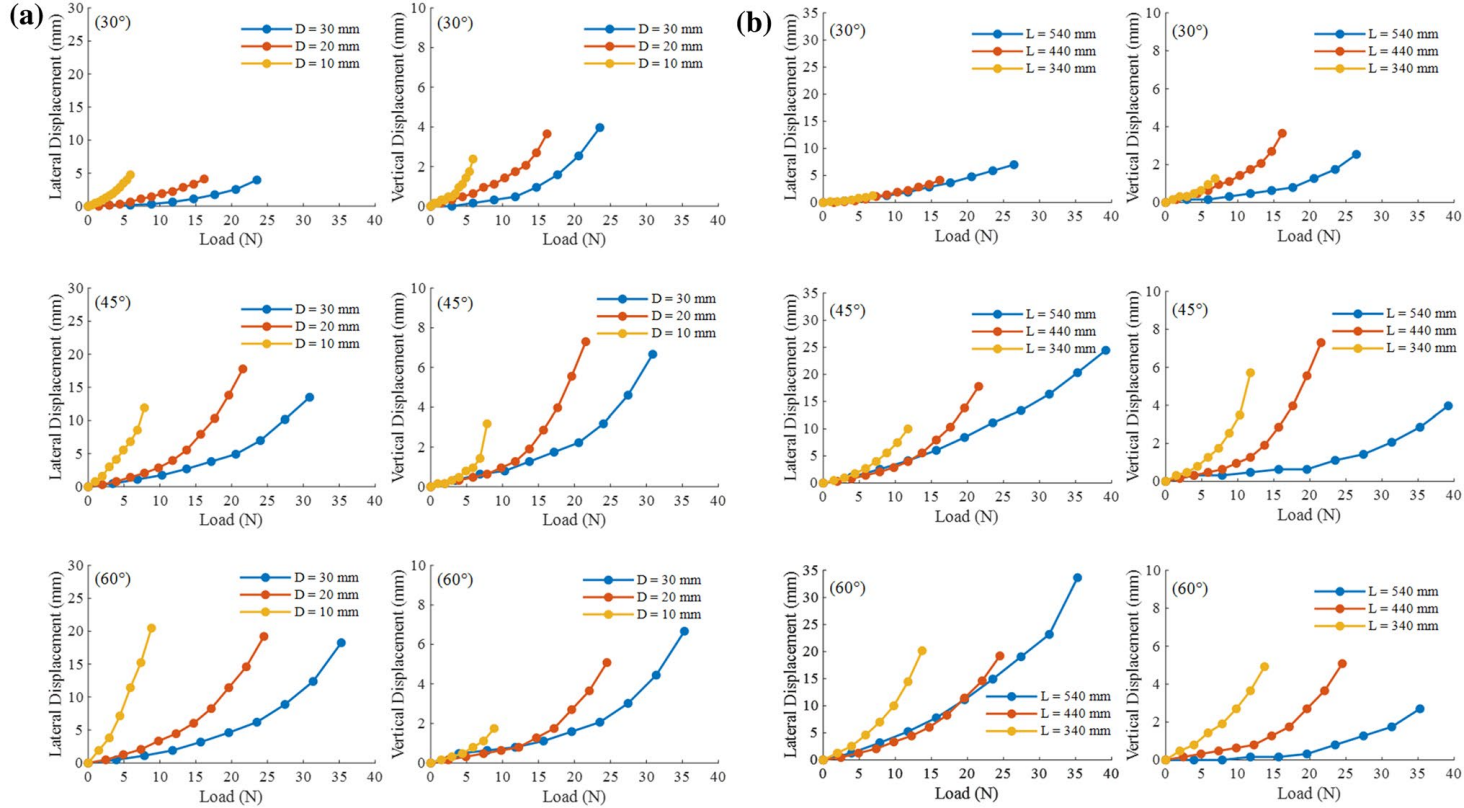
Lateral and vertical displacement–load curve of circular single pile’s with variables in pile diameter (**a**) and embedded depth (**b**).

Effect of the pile diameter on the uplift resistance capacity of a circular single pile is shown in Fig. 7a. The figure shows the displacement–load curves of circular single piles with variables pile diameter (D = 10, 20, and 30 mm) at the initial tension angles of 30°, 45°, and 60° (Case 3). As the pile diameter increased, the slope of the lateral and vertical displacement–load curve of the pile top gradually decreased, and the curve became smoother. This implied that increasing the pile diameter effectively improved the uplift resistance capacity of the anchor pile. There was no significant difference in the maximum lateral displacement for variables pile diameter at the same tension angle. The vertical displacement–load curves show that the maximum vertical displacement for the D = 10 mm test case is significantly smaller than those for the D = 20 and 30 mm test cases, and the maximum vertical displacements of the D = 20 and 30 mm test cases were not significantly different. In summary, the increase in pile diameter can effectively delay the development speed of displacement but has little effect on the maximum displacement.

**Figure 8:**
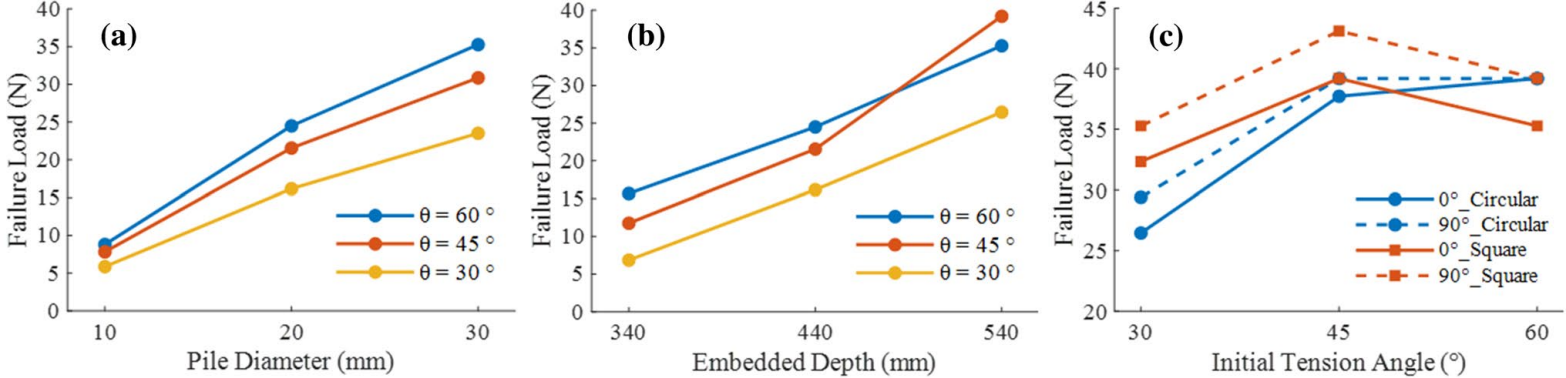
Failure loads of anchor pile models with variables in pile diameter (**a**), embedded depth (**b**) and pile configuration (**c**).

Figure 8a shows a comparison of the failure loads of the anchor piles for variables pile diameter. The failure load of the anchor piles increased as the pile diameter increased. When $\theta$ = 30°, 45°, and 60°, the failure loads of the D = 10 mm test case were 5.88, 7.84, and 8.82 N, respectively. The failure loads of the D = 20 mm test case were 16.17, 21.56, and 24.5 N, and compared to those of the 10 mm pile diameters, the failure loads increased by 175.0$\%$, 175.0$\%$, and 177.8$\%$, respectively. The failure loads of D = 30 mm were 23.52, 30.87, and 35.28 N, respectively, which are 45.4$\%$, 43.2$\%$, and 44$\%$ higher than those of D = 20 mm Consistent with the literature^31^. Wang et al.^32^ concluded that as the pile diameter increases, when the pile fails, the soil will change from a shallow wedge failure to a combination of shallow wedge failure and deep rotating soil flow. Therefore, the anchor pile uplift resistance capacity will not increase with the increase in pile diameter by an equal margin. Although the number of pile diameter samples in this experiment is not sufficient, the improvement of D=30mm compared to D=20mm is significantly less than the improvement of D=20mm compared to D=10mm when comparing the value of the failure load.

### Effect of embedded depth on uplift resistance capacity

Effect of the embedded depth on the uplift resistance capacity of a circular single pile is shown in Fig. 7b. The figure shows the displacement–load curves of the anchor piles with variables embedded depth (L = 340, 440, and 540 mm) at the initial tension angles of 30°, 45°, and 60° (Case 4). The figure shows that for the three initial tension angles, the displacement–load curves of variables embedded depth differ significantly. From the lateral displacement curves, the curve for L = 340 mm developed the fastest among the three cases. For small loads, the curves of L = 440 mm and L = 540 mm have similar slopes, and the curves partially overlap. As the load increased, the anchor piles for L = 440 mm fail, and the displacement increased rapidly. The curves of L = 440 mm and L = 540 mm appear to be different. From the vertical displacement–load curve, the curve slopes gradually decrease with an increase in the embedded depth. The displacement development speed of the L = 540 mm test case was the slowest. Increasing the embedded depth effectively delayed the development speed of vertical displacement.

Ai et al.^33^ found that increasing the embedded depth could not effectively limit the development of lateral displacement. In this experiment, except for the L = 340 mm case when $\theta$ = 60°, the maximum lateral displacement may be slightly larger than the L = 440 mm case because of experimental errors. In other cases, the maximum lateral displacement at the top of the pile increases with raising embedment depth, with a maximum at L = 540 mm. The maximum vertical displacement for L = 440 mm is the largest among the three embedded depths, and the maximum vertical displacement for L = 540 mm is the smallest.

Figure 8b shows the relationship between the anchor pile failure load and the embedded depth under variables initial tension angle. The figure shows that the failure load increased with an increase in embedded depth. When $\theta$ = 30°, 45°, and 60°, the failure loads of L = 340 mm were 6.86, 11.76, and 15.68 N, respectively. The failure loads of L = 440 mm were 16.17, 21.56, and 24.50 N, respectively, and compared with L = 340 mm, increased by 135.7$\%$, 83.3$\%$, and 56.3$\%$, respectively. The failure loads of L = 540 mm were 26.46, 39.2, and 35.28 N, respectively, and compared with L = 440 mm, increased by 63.6$\%$, 81.8$\%$, and 44$\%$, respectively. Gaaver et al.^34^ found two main explanations for why the embedded depth improves the uplift resistance. 1. Improved friction in the pile–soil interface. Thus, the increased embedded depth increases the effective stress in the anchor pile and the soil shear strength. 2. Increased contact area between the anchor pile and soil. Emirler et al.^35^ used numerical simulations to study the uplift resistance capacity of piles with variables embedded depth in sand and found that the area of effect of the anchor pile on the sand surface increased with an increase in the embedded depth. Although in this study, the effect of the anchor pile under oblique load differs slightly from the above research, increasing the embedded depth still increases the uplift resistance capacity of the anchor pile.

In this experiment, the failure load of L = 540 mm test case, $\theta$ = 60° is less than $\theta$ = 45°, which is a special phenomenon but not a coincidence. With the raising of embedment depth, the lateral clay reaction force increases, and when the load reaches the limit of lateral clay reaction force, the clay displacement is too large leading to the premature failure of anchor pile and the decrease of uplift resistance. Jamnejad et al.^36^ conducted experimental and theoretical studies on anchor piles installed vertically in saturated non-cohesive clay and concluded that the uplift resistance capacity of anchor piles under oblique loads would increase with embedded depth. When the embedded depth reaches a certain level, a further increase in the embedded depth can only lead to an increase in the vertical component, and the effect on the lateral component and the improvement in the uplift resistance capacity are no longer significant.

### Effect of pile configuration on the uplift resistance capacity

In marine aquaculture, two anchor piles are often tied together to improve the uplift resistance capacity of the anchor piles. In this section, two-pipe anchor pile models were posted side-by-side using plexiglass glue to simulate a two-pipe anchor pile. The dual anchor pile model used in this experiment is shown in Fig. 3c. To study the effect of pile configuration on the resistance to uplift resistance capacity, dual piles are installed using two methods. Figure 10a,b illustrates the specific situation. 0° and 90° represent the angle between the dual pile model and the tension direction.

**Figure 9:**
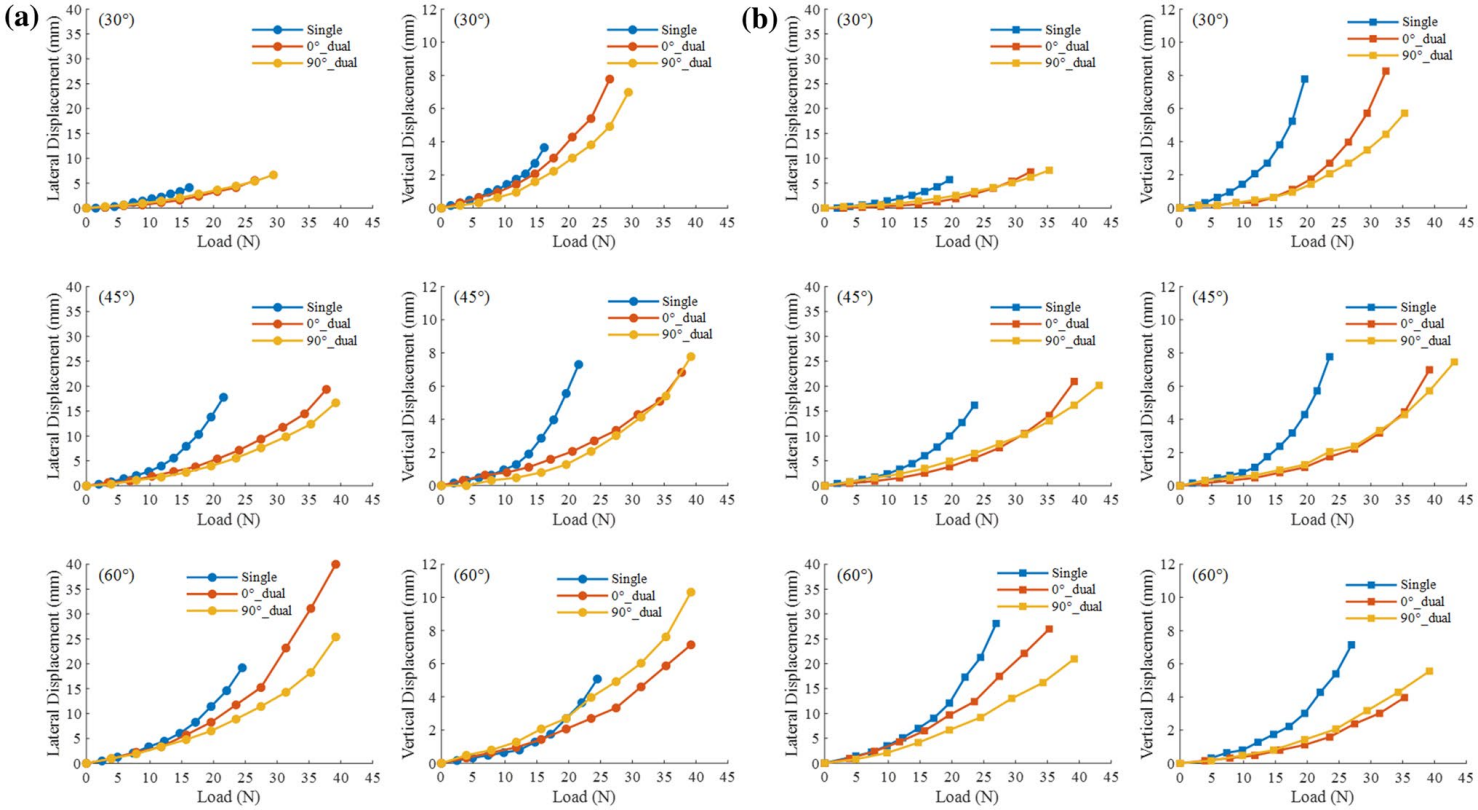
Lateral and vertical displacement-load cures of circular (**a**) and square (**b**) anchor piles with variables configuration.

Figure 9 show the displacement–load curves for variables pile configuration of circular and square anchor piles (single pile, 0°$\_$dual layout, 90°$\_$dual layout) under the initial tension angles of 30°, 45°, and 60° (Cases 5 and 6). The figures show that the development speed of the lateral and vertical displacements of the dual pile was significantly lower than that of the single pile. Table 4 is the maximum displacement of pile top under variables configuration. In conjunction with Fig. 9 and Table 4 to analysis. The lateral displacement of the 0°$\_$dual layout was greater than that of the 90°$\_$dual layout, and the vertical displacement of the 90°$\_$dual layout was greater than that of the 0°$\_$dual layout. The displacement development and maximum displacement of the square anchor piles under variables pile configuration were similar to those of the circular anchor piles.

**Figure 10:**
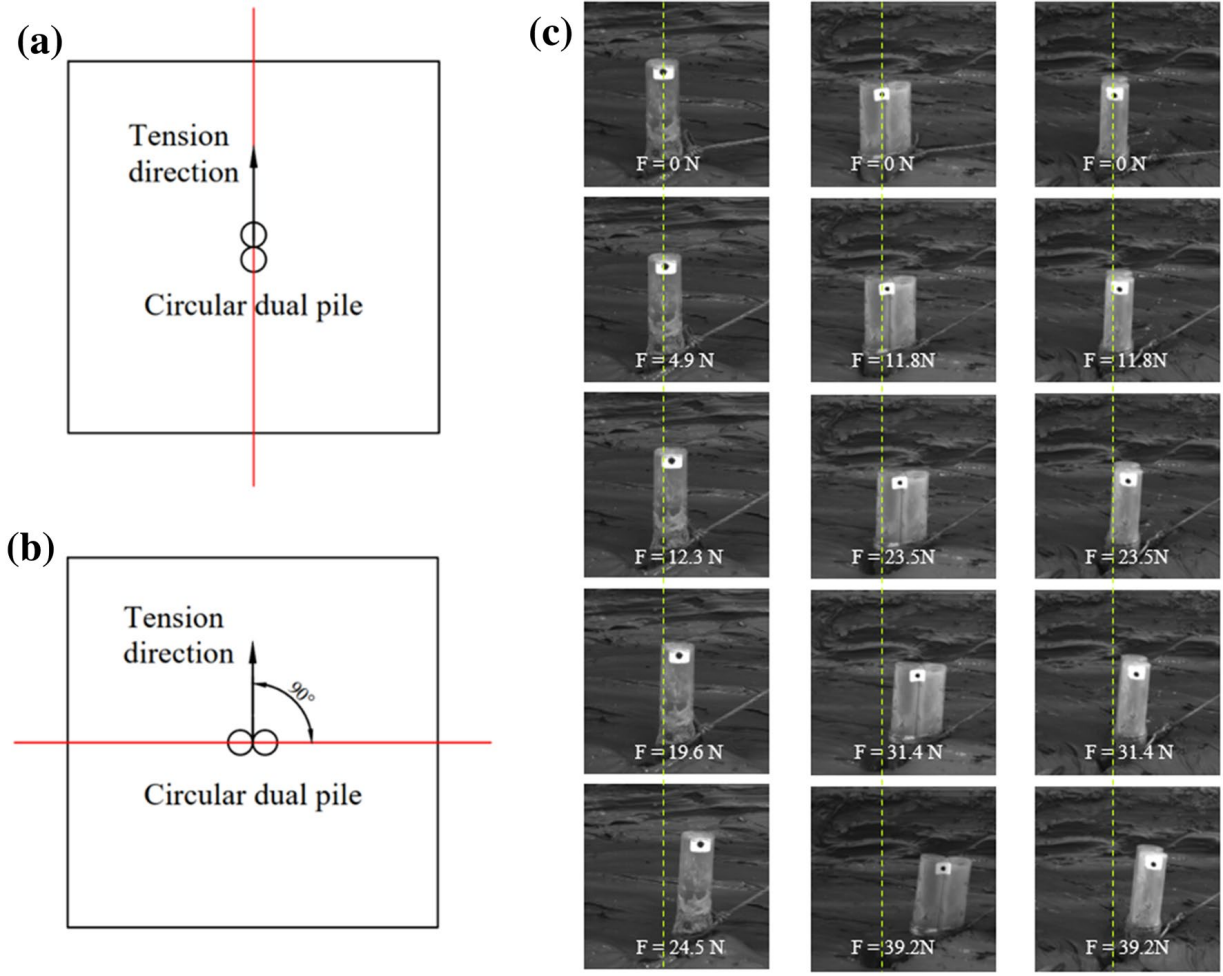
Different layouts of dual piles 0°$\_$dual (**a**) and 90°$\_$dual (**b**). Photos of the failure process of variables pile configuration (left column: circular single pile; middle column:circular 0°$\_$dual layout; right column: circular 90°$\_$dual layout) with $\theta$ = 60° (**c**).

Figure 10c demonstrates the differences in pile configurations more intuitively. It shows photographs of the destruction process of variables pile configuration. The figure shows that the load required for the same displacement of the circular single pile was less than that of the dual pile. When the applied load F was the same, the displacement of the circular 90°$\_$dual layout was smaller than that of the 0°$\_$dual layout. In this experiment, the lateral displacement of 90°$\_$dual is significantly less than 0°$\_$dual when $\theta$ = 60°.

**Table 4:** Maximum displacement of pile top under variables configuration.

Layout angle (°)	Initial tension angle (°)	Circular dual pile			Square dual pile		
		Lateral displacement (mm)	Vertical displacement (mm)	Failure load (N)	Lateral displacement (mm)	Vertical displacement (mm)	Failure load (N)
0	30	5.56	7.78	26.46	7.3	8.25	32.34
	45	19.37	6.83	37.73	20.95	6.98	39.2
	60	40	7.14	39.2	26.98	3.97	35.28
90	30	6.67	6.98	29.4	7.62	5.71	35.28
	45	16.67	7.78	39.2	20.16	7.46	43.12
	60	25.4	10.32	39.2	20.95	5.56	39.2

Figure 8c shows the relationship between the failure load of the double-pipe anchor pile and the initial tension angle. The figure shows that the uplift resistance capacity of the 90°$\_$dual layout was better than that of the 0°$\_$dual layout. Reddy et al.^14^ concluded that when the oblique tension load was too large, the lateral reaction force of the clay around the anchor pile was insufficient, and the anchor pile was destroyed prematurely, resulting in a reduction in the failure load. This could be the reason for the decrease in the failure load of the square dual pile at $\theta$ = 60° in this experiment. In addition, in the experiment discussed in last section on the effect of the embedded depth on the uplift resistance capacity, the embedded depth of 540 mm coincides with the maximum failure load in the case of $\theta$ = 45°. This shows that in marine aquaculture, the best initial tension angle of anchor piles is not as large as possible, and factors such as clay quality and tube type should be comprehensively considered.

## Conclusions

In this study, by performing oblique anchor pile uplift experiments on models, the effects of initial tension angle, pile type, pile diameter, embedded depth, and pile configuration on the uplift resistance capacity of anchor piles were determined. Below are the main conclusions:

- Circular and square single pile, both the uplift resistance capacity and the anchor pile top displacement development speed increased with an increase in the initial tension angle. When the initial tension angle was greater than 45°, the lateral displacement drastically increased.
- The uplift capacity of square single pile is slightly better than that of circular single pile due to the larger contact area between the square section and the clay although the side length of the square single pile equals the diameter of the circular single pile.
- The increase in the pile diameter can effectively improve the uplift resistance capacity of the circular anchor pile and delay the development speed of the pile top displacement.
- The increase in the embedded depth can effectively improve the uplift resistance capacity of the circular anchor pile and increase the maximum lateral displacement of the pile top before failure.
- The uplift resistance capacity of the dual pile was significantly better than that of the single pile. The uplift resistance capacity of the 90°$\_$dual layout was slightly better than that of the 0°$\_$dual layout, but the improvement was not significant. 90°$\_$dual layout is effective in reducing lateral displacement when $\theta>$ 45°.

## References

[1] Zhao Y-P (2019). Hydrodynamic responses of longline aquaculture facility with lantern nets in waves. Aquac. Eng..

[2] Bi C-W, Zhao Y-P, Dong G-H, Xu T-J, Gui F-K (2014). Numerical simulation of the interaction between flow and flexible nets. J. Fluids Struct..

[3] Feng D, Meng A, Wang P, Yao Y, Gui F (2021). Effect of design configuration on structural response of longline aquaculture in waves. Appl. Ocean Res..

[4] Zhao Y-P, Bai X-D, Dong G-H, Bi C-W (2019). Deformation and stress distribution of floating collar of net cage in steady current. Ships Offshore Struct..

[5] Trujillo E, León L, Martínez G (2020). Deadweight anchoring behavior for aquaculture longline. Latin Am. J. Aquat. Res..

[6] Cortes-Garcia LD, Landon ME, Gallant AP, Huguenard KD (2019). Assessment of Helical Anchor Capacity in Marine Clays for Aquaculture Applications.

[7] Hou H-M, Dong G-H, Xu T-J, Zhao Y-P, Bi C-W (2018). Dynamic analysis of embedded chains in mooring line for fish cage system. Pol. Marit. Res..

[8] Gaudin C (2017). Recent advances in anchor design for floating structures. Int. J. Offshore Polar Eng..

[9] Rao SN, Latha KH, Pallavi B, Surendran S (2006). Studies on pullout capacity of anchors in marine clays for mooring systems. Appl. Ocean Res..

[10] Esfeh PK, Kaynia AM (2019). Numerical modeling of liquefaction and its impact on anchor piles for floating offshore structures. Soil Dyn. Earthq. Eng..

[11] Yi JT (2020). Pull-out capacity of an inclined embedded torpedo anchor subjected to combined vertical and horizontal loading. Comput. Geotech..

[12] Cerfontaine B, Knappett J, Brown MJ, Davidson C, Sharif Y (2020). Optimised design of screw anchors in tension in sand for renewable energy applications. Ocean Eng..

[13] Ayothiraman, R. & Reddy, K. M. Model experiments on pile behaviour in loose-medium dense sand under combined uplift and lateral loads. *In Tunneling and Underground Construction*, **GSP 242,** 633–643 (2014).

[14] Madhusudan Reddy K, Ayothiraman R (2015). Experimental studies on behavior of single pile under combined uplift and lateral loading. J. Geotech. Geoenviron. Eng..

[15] Lu W, Zhang G (2018). Influence mechanism of vertical-horizontal combined loads on the response of a single pile in sand. Soils Found..

[16] Shin E, Das B, Puri V, Yen S, Cook E (1993). Ultimate uplift capacity of model rigid metal piles in clay. Geotech. Geol. Eng..

[17] Yang Mh, Yang Xw, Zhao Mh (2016). Study of model experiments on uplift piles in clay under oblique loads. J. Hunan Univ. (Nat. Sci.).

[18] Ramadan MI, Butt SD, Popescu R (2013). Offshore anchor piles under mooring forces: Centrifuge modeling. Can. Geotech. J..

[19] Ramadan MI, Butt SD, Popescu R (2013). Offshore anchor piles under mooring forces: Numerical modeling. Can. Geotech. J..

[20] Hu C-B, Mei L, Mei G-X, Zai J-M (2009). Finite element method for selecting the soil boundary in the model of pile-soil. Build. Sci..

[21] Saravanan R, Arumairaj P, Subramani T (2018). A study on behavior of vertical pile in sand under uplift load. Geotech. Eng. (SEAGS & AGSSEA).

[22] Sedov LI (1993). Similarity and Dimensional Methods in Mechanics.

[23] Bhardwaj S, Singh S (2015). Pile capacity under oblique loads-evaluation from load-displacement curves. Int. J. Geotech. Eng..

[24] Qiu Y, Gao YF, Bing LI, Wang YK, Di WU (2017). Calculation methods for ultimate inclined bearing capacity of caisson foundation under inclined load. J. Yangtze River Sci. Res. Inst..

[25] Bhardwaj S, Singh S (2015). Influence of load obliquity on pullout capacity of micropile in sand. Indian Geotech. J..

[26] Renpeng KLJLC, Yunmin FJLGC (2013). Response of squeezed branch piles under inclined uplift loads. Chin. J. Appl. Mech..

[27] Gao Y (2013). Experimental studies on the anti-uplift behavior of the suction caissons in sand. Appl. Ocean Res..

[28] Achmus M, Thieken K (2010). On the behavior of piles in non-cohesive soil under combined horizontal and vertical loading. Acta Geotech..

[29] Conte E, Troncone A, Vena M (2015). Behaviour of flexible piles subjected to inclined loads. Comput. Geotech..

[30] Wen S-L, Wang X-B (2012). Numerical simulation on the impact of loading angle on the bearing characteristics of pile under the inclined load. Build. Sci..

[31] Al-Mhaidib AI, Edil TB (1998). Model tests for uplift resistance of piles in sand. Geotech. Test. J..

[32] Wang H, Wang L, Hong Y, He B, Zhu R (2020). Quantifying the influence of pile diameter on the load transfer curves of laterally loaded monopile in sand. Appl. Ocean Res..

[33] Ai ZY, Zhao YZ, Cheng YC (2020). Time-dependent response of laterally loaded piles and pile groups embedded in transversely isotropic saturated viscoelastic soils. Comput. Geotech..

[34] Gaaver, Khaled E (2013). Uplift capacity of single piles and pile groups embedded in cohesionless soil. Alex. Eng. J..

[35] Emirler B, Tolun M, Yildiz A (2019). 3d numerical response of a single pile under uplift loading embedded in sand. Geotech. Geol. Eng..

[36] jamnejad G, Hesar M, de Souza C, Hobbs R, Price J (1995). Stability of pile anchors in the offshore 323 environment. discussion. Transactions-Institute Mar. Eng..

